# Versatile β-Catenin Is Crucial for SARS-CoV-2 Infection

**DOI:** 10.1128/spectrum.01670-22

**Published:** 2022-08-31

**Authors:** Sanchari Chatterjee, Supriya Suman Keshry, Soumyajit Ghosh, Amrita Ray, Soma Chattopadhyay

**Affiliations:** a Institute of Life Sciences, Bhubaneswar, Odisha, India; b Regional Centre for Biotechnology, Faridabad, Haryana, India; c School of Biotechnology, Kalinga Institute of Industrial Technology (KIIT) University, Bhubaneswar, Odisha, India; Indian Institute of Science Bangalore

**Keywords:** SARS-CoV-2, COVID-19, β-catenin, Wnt/β-catenin pathway, iCRT14

## LETTER

**FIG 1:**
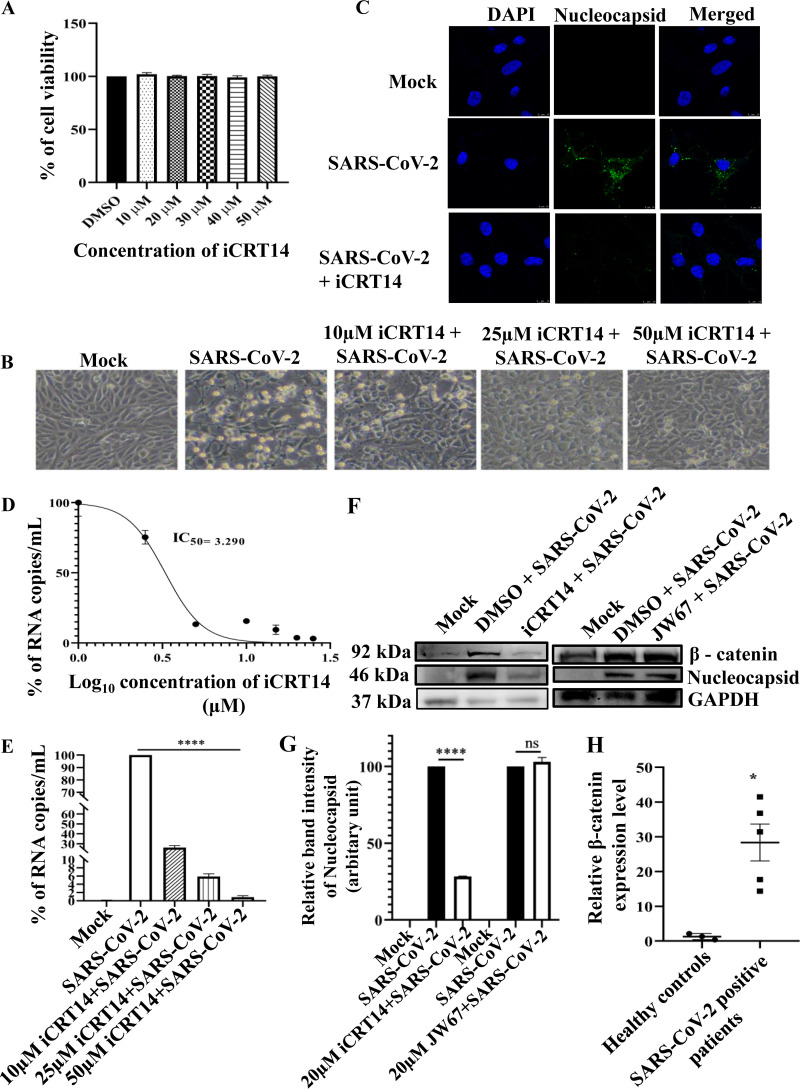
The β-catenin protein was crucial for SARS-CoV-2 infection. Vero cells were treated with different concentrations (10, 20, 30, 40, or 50 μM) of iCRT14 for 15 h and the cytotoxicity of the cells was estimated by MTT assay. (A) Bar diagram showing the viability of cells. (B) Images representing the CPE of cells that were observed under a microscope (20× magnification) with or without the iCRT14 treatment. (C) Immunofluorescence analysis revealing mock or infected Vero cells stained with nucleocapsid antibody. Nuclei were counterstained with 4,6-diamidino-2-phenylindole (DAPI). Scale bar = 10 μm. (D) The IC_50_ of iCRT14 against SARS-CoV-2 virus in Vero cells was estimated. The *x*-axis illustrates the logarithmic value of the different concentrations of iCRT14, and the *y*-axis portrays the percentage of RNA copies/mL. (E) Viral RNA was isolated from the mock, SARS-CoV-2 infected and different concentrations (10, 25, or 50 μM) of iCRT14 treated cells, and the SARS-CoV-2 nucleocapsid gene was amplified by RT-qPCR. Bar diagram exhibiting the percentage of viral RNA copies/mL. (F) Western blot showing the nucleocapsid and β-catenin proteins of mock, infected, JW67, and iCRT14 treated infected cells. Glyceraldehyde 3-phosphate dehydrogenase (GAPDH) served as the loading control. (G) Bar diagram representing the relative band intensity of the nucleocapsid protein. (H) Bar diagram depicting relative β-catenin expression level after normalizing with RNaseP, from healthy control and SARS-CoV-2 infected patients. Data from three independent experiments are shown as mean ± SEM. *, *P* < 0.05; **, *P* < 0.01; ***, *P* < 0.001, and ****, *P* < 0.0001 were considered statistically significant.

**FIG 2:**
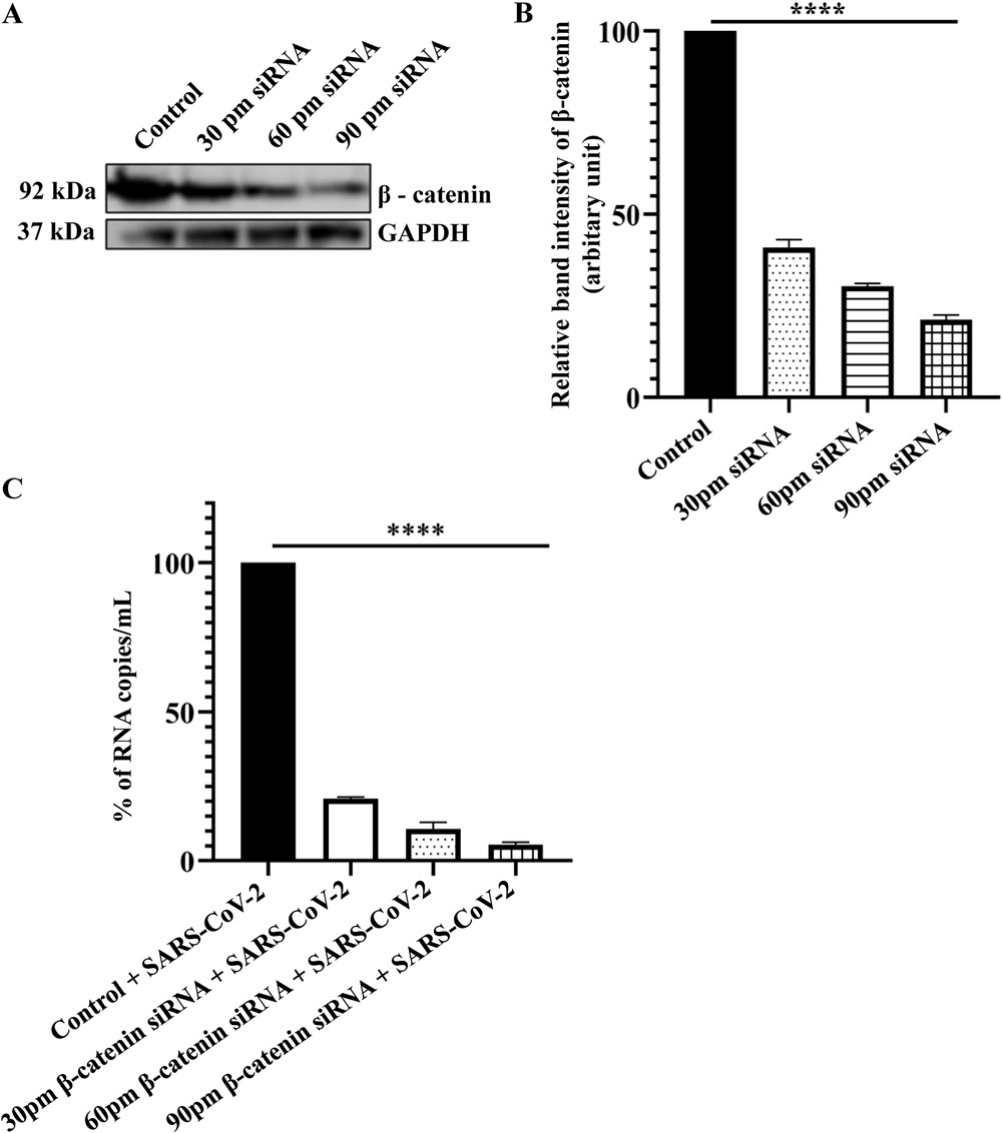
Knockdown of β-catenin reduced SARS-CoV-2 infection. Vero cells were transfected with 30, 60, or 90 pm of β-catenin siRNA. The transfected cells were superinfected with SARS-CoV-2 after 24 hpt and harvested for RT-qPCR. (A) The β-catenin level was estimated from the Western blot, and GAPDH was used as a loading control. (B) Bar diagram showing relative band intensity of β-catenin protein. (C) Bar diagram exhibiting the viral RNA copy number/mL in the cell supernatant of control + SARS-CoV-2 infected and β-catenin siRNA+ SARS-CoV-2 infected samples. Data of three independent experiments are shown as mean ± SEM. *, *P* < 0.05; **, *P* < 0.01; ***, *P* < 0.001, and ****, *P* < 0.0001 were considered statistically significant.

The recent pandemic caused by the severe acute respiratory syndrome coronavirus 2 (SARS-CoV-2) led to the loss of lives at an unprecedented scale. In 2022, it is continuing to baffle world health at large. Several intervention strategies, including drug repurposing, were done to combat the virus. Still, the emergence of new variants (1) continues to question the methods of interference. All the viruses exploit a plethora of tactics to modulate the host signaling to aid their survival (2). Hence, in the current investigation, the host factors whose modulation might prove to be a much more effective strategy were explored. Interestingly, β-catenin, a key component of the Wnt/β-catenin pathway, was found to be crucial for SARS-CoV-2 replication. To verify the importance of the β-catenin protein in SARS-CoV-2 infection, iCRT14, a specific inhibitor of the Wnt/β-catenin signaling pathway, was used. The cytotoxicity of the iCRT14 in Vero cells was checked by an 3-(4,5-dimethylthiazol-2-yl)-2,5-diphenyl tetrazolium bromide (MTT) assay, and it was found that 100% of cells were viable at all the concentrations of the inhibitor (Fig. 1A). After SARS-CoV-2 infection and iCRT14 treatment (10, 25 and 50 μM), cytopathic effect (CPE) was observed under a microscope. A visible decrease in CPE was seen after iCRT14 treatment in a dose-dependent manner compared to the dimethyl sulfoxide (DMSO) control (Fig. 1B). The reduction in infection was further correlated with the Immunofluorescence assay, which revealed a drastic reduction of the SARS-CoV-2 nucleocapsid protein level in iCRT14 treated cells as shown in Fig. 1C. For the determination of the IC_50_ of iCRT14, the cells were treated with different concentrations of the drug after infection and the supernatants were subjected to viral RNA isolation and real-time quantitative PCR (RT-qPCR). The viral copy number was determined by generating the standard curve of the SARS-CoV-2 nucleocapsid gene. The percentage of copy number/mL was calculated from the corresponding cycle threshold (Ct) values of all the samples (3). The graph for the IC_50_ was plotted using the GraphPad Prism software (4). The IC_50_ of iCRT14 was found to be 3.2 μM against the SARS-CoV-2 virus (Fig. 1D). Additionally, the effect of 10, 25, and 50 μM concentrations of iCRT14 was checked on SARS-CoV-2 viral copy numbers from virus-infected and iCRT14-treated cells. RT-qPCR data revealed a 73.9%, 94.2%, and 99.1% reduction in the SARS-CoV-2 viral copy numbers in the culture supernatants (Fig. 1E). To strengthen the results, JW67, an inhibitor of the Tankyrase protein of Wnt signaling has been used. This inhibitor can accumulate inactive β-catenin in the cytoplasm (5) and did not influence the replication of SARS-CoV-2. Thus, a Western blot showed that JW67 did not reduce β-catenin and nucleocapsid proteins in comparison to DMSO + SARS-CoV-2 control, while iCRT14 treatment showed a drastic reduction of the SARS-CoV-2 nucleocapsid and β-catenin proteins (Fig. 1F and G). Interestingly, a higher expression level of β-catenin was found in SARS-CoV-2 infected patients (*n* = 5), compared to the healthy controls (*n* = 3, Fig. 1H). The Institutional Ethics Committee (IEC)/Institutional Review Board (IRB) reference number is 96/HEC/2020. Written consent informed to participate in this study was provided by the participant's legal guardian/next of kin. These results indicated the importance of β-catenin protein for SARS-CoV-2 infection. To further validate the results, siRNA concentrations of 30, 60, and 90 pm were used to silence the expression of the β-catenin gene in Vero cells. Twenty-four hours posttransfection (hpt), cells were harvested and processed for Western blotting. It was observed that the β-catenin protein level was reduced by 59.1%, 69.7%, and 78.9% using 30, 60, and 90 pm concentrations of siRNA, respectively, compared to control (Fig. 2A and B). Next, the siRNA transfected cells were superinfected with SARS-CoV-2, and supernatants were processed for RT-qPCR to assess the level of the nucleocapsid. Interestingly, it was found that there was a 79.1%, 89.3%, and 94.6% reduction in the viral copy number in comparison to the control (Fig. 2C). Taken together, it was established that β-catenin was crucial for efficient SARS-CoV-2 infection. However, the actual mechanism of its modulation during viral infection has yet to be explored. The Wnt/β-catenin pathway has been modulated by various viruses. Earlier reports suggested that members of the miR-34 family, which can repress Wnt/beta-catenin signaling, demonstrated strong anti-flaviviral effects (6). It was also observed that knockdown of β-catenin reduced the Rift Valley fever virus (7).

In summary, the current study identified the crucial role of the β-catenin protein belonging to the canonical Wnt/β-catenin pathway for SARS-CoV-2 infection, which may help to design efficient therapeutics to curb the SARS-CoV-2 infection in the future.
